# Inhibition of valve mesenchymal stromal cell calcium deposition by bFGF through alternative polyadenylation regulation of the *CAT* gene

**DOI:** 10.1186/s12872-024-03775-5

**Published:** 2024-02-28

**Authors:** Jiajun Zhang, Jun Wu, Yuan Gao, Xingli Fan, Xiaohong Liu, Guanxin Zhang, Yangfeng Tang, Lin Han

**Affiliations:** Department of Cardiovascular Surgery, Changhai Hospital, Second Military Medical University, 168 Changhai Road, Shanghai, 200433 China

**Keywords:** Calcific aortic valve disease, Alternative polyadenylation, Catalase, bFGF

## Abstract

**Objective:**

Calcific aortic valve disease (CAVD) is the leading cause of angina, heart failure, and death from aortic stenosis. However, the molecular mechanisms of its progression, especially the complex disease-related transcriptional regulatory mechanisms, remain to be further elucidated.

**Methods:**

This study used porcine valvular interstitial cells (PVIC) as a model. We used osteogenic induced medium (OIM) to induce calcium deposition in PVICs to calcify them, followed by basic fibroblast growth factor (bFGF) treatment to inhibit calcium deposition. Transcriptome sequencing was used to study the mRNA expression profile of PVICs and its related transcriptional regulation. We used DaPars to further examine alternative polyadenylation (APA) between different treatment groups.

**Results:**

We successfully induced calcium deposition of PVICs through OIM. Subsequently, mRNA-seq was used to identify differentially expressed mRNAs for three different treatments: control, OIM-induced and OIM-induced bFGF treatment. Global APA events were identified in the OIM and bFGF treatment groups by bioinformatics analysis. Finally, it was discovered and proven that *catalase* (*CAT*) is one of the potential targets of bFGF-induced APA regulation.

**Conclusion:**

We described a global APA change in a calcium deposition model related to CAVD. We revealed that transcriptional regulation of the *CAT* gene may contribute to bFGF-induced calcium deposition inhibition.

**Supplementary Information:**

The online version contains supplementary material available at 10.1186/s12872-024-03775-5.

## Introduction

Calcific aortic valve disease (CAVD) is a prevalent valvular heart disease in the aging population and is characterized by the thickening of the aortic valve leaflets and nonrheumatic calcium deposition [1, 2]. The symptoms of CAVD are minimal within the progression range of aortic sclerosis (mild valve thickening without the obstruction of blood flow) and aortic stenosis (severe calcium deposition with impaired leaflet motion) [2]. However, pressure overload may eventually lead to progressive left ventricular hypertrophy, angina, syncope, and heart failure. Valvular interstitial cells (VICs) constitute the major cell population in the aortic heart valve [3] and are responsible for maintaining valve homeostasis. VICs from porcine hearts have been established as a classic model for studying the pathological stimuli involved in CAVD [1].

Despite the huge efforts devoted to CAVD research, effective medical therapies to halt CAVD progression as an alternative to surgery are lacking. Next-generation sequencing-based RNA-Seq has the potential to identify the regulatory mechanisms of CAVD initiation and progression by investigating the continuously changing cellular transcriptome. Schlotter et al. identified the first molecular regulatory networks in CAVD by combining RNA-seq and proteomics [4]. Identification of the contribution of endothelial-to-mesenchymal transition in CAVD pathogenesis was achieved by combining both bulk RNA-seq and scRNA-seq [5].

Alternative polyadenylation (APA) is a major post-transcriptional regulatory mechanism that generates alternative 3′ termini on mRNAs and other RNA polymerase II transcripts [6]. APA is widespread across all eukaryotic species, and occurs most frequently in the 3′ untranslated region (3′ UTR) of mRNAs [7]. Basic fibroblast growth factor (bFGF) is a heparin-binding growth factor family protein that functions in coordination with another growth factor, epidermal growth factor (EGF), to regulate cell proliferation, osteogenesis, and other basic cellular processes [8, 9]. Recent studies have shown that increased APA events are at least partially responsible for the pro-survival effect of EGF [10]. bFGF is an effective inhibitor of calcium deposition [11]. Therefore, we aimed to investigate whether bFGF and calcium deposition treatment can lead to changes in APA in porcine valvular interstitial cells (PVICs), thus affecting the total transcriptomic profiles. We performed mRNA-seq on bFGF and osteogenic induced medium (OIM) -treated PVICs to reveal the transcriptome changes in PVICs after treatment compared to those of untreated cells. Our results demonstrate, for the first time, the APA changes in bFGF- and OIM-treated PVICs. We propose that careful manipulation of APA in catalase (*CAT*) may be useful in rescuing calcium deposition in PVICs, thus treating CAVD.

## Materials and methods

### Cell culture and treatments

Porcine aortic valves were purchased from a local slaughterhouse and transported in ice-chilled glucose. Dulbecco’s modified Eagle’s medium (DMEM) with 10% fetal bovine serum (FBS, Gibco Laboratories, Gaithersburg, MD, USA) and amphotericin B (1.25 μg/mL) for cell culture. Porcine aortic valve specimens were cut into small pieces after washing six times in PBS with 1% penicillin/streptomycin and digested in type I collagenase (2 mg/mL) (Sigma-Aldrich, Saint Louis, MO, USA) at 37 °C for 8–10 h in an atmosphere containing 5% CO2. Subsequently, a 70 μm nylon mesh strainer was used to remove undigested tissue, and the separated cells were seeded in DMEM supplemented with 10% FBS and 1% penicillin/streptomycin at 37 °C under 5% CO2 for primary cultures.

The culture medium was changed every 3 days. Third- or fourth-generation cells were used for all experiments [12]. For the osteogenic differentiation model of PVICs, the cells were cultured in OIM (Cyagen Biosciences, HUXMA-90021, complete medium supplemented with 50 μg/mL ascorbic acid, 2 mmol/L sodium dihydrogen phosphate dihydrate, and 10–7 mol/L insulin, changed every 2–3 days.) to stimulate osteogenic differentiation according to previously described protocols [13] after the cells reached 78–80% confluence. Untreated interstitial cells cultured in DMEM were used as the negative control. All samples were assayed in triplicates. bFGF (50 ng/mL, Peprotech, Cranbury, NJ, USA) was added to the OIM medium for subsequent treatment. The VICs were seeded in 6- well plate, and the total RNA was extracted 48 hours after each treatment. Trizol was added to each well to lyse the VICs after being washed 3 times with PBS.

### Calcium deposition analysis

Cells were cultured in 6-well plates, and specific OIM and bFGF treatments were performed until reaching 85% confluence. After treatment for 7 days, Alizarin red staining was performed to evaluate the degree of cell calcium deposition. Briefly, cells were washed twice in PBS, then fixed in 4% formaldehyde for 15 min, and stained with 0.1% Alizarin Red solution for 30 min at room temperature. The red color indicated calcified nodule formation, which was detected under a microscope at × 400 magnification.

### RNA extraction and quality control

Total RNA was extracted using TRIzol RNA reagent (Life Technologies, Cat. # 15596–018, Carlsbad, CA, USA) according to the manufacturer’s instructions. RNA integrity and concentration were determined using a NanoDrop ND-1000 Spectrophotometer (Thermo Fisher Scientific, Waltham, MA, USA). Total RNA was further purified using a RNeasy mini kit (QIAGEN, Cat. # 74106, GmBH, Hilden, Germany), and RNase-free DNase set (QIAGEN, Cat. # 79254, GmBH).

### RNA-seq analysis

The RNA-seq library was prepared using NEBNext Ultra RNA with Poly-A selection and sequenced on an Illumina Hi-Seq 4000. After running fastQC (0.11.8) for quality control, Cutadapt (2.1.0) was used to trim adapters and low-quality sequences (Phred score less than 20) from raw fastq files. The cleaned fastq files were then mapped to the susScr11 reference genome using STAR (2.7.0) with the default settings. Differential expression analysis was performed using DEseq2 (version 1.29.0). Significant differentially expressed genes (DEGs) were defined by a *p*-value ≤0.05 [false discovery rate (FDR) correction] and a log_2_ fold change ≥0.5. Pathway enrichment analysis was performed using the EnrichR software. The bioconductor volcano plot was generated using R (version 3.5.0). Gene ontology (GO) analysis was performed using the clusterprofiler package from Bioconductor.

### APA detection and pathway enrichment

The RNA-seq wig file generated using STAR was used as the input for DaPars (0.9.1) [14] to identify APA events. Significant events were defined by a *p*-value < 0.05 and an absolute delta distal polyA site usage index (PDUI) > 0.1. GO analysis was performed to determine the roles of these differentially expressed 3’UTRs. GO analysis revealed three major areas: biological process (BP), cellular component (CC), and molecular function (MF). Kyoto Encyclopedia of Genes and Genomes (KEGG) analysis was used to identify the enriched biochemical pathways involving the DEGs. Pathways with *p*-value less than 0.05 were considered as significantly enriched pathways.

### Qualitative real-time PCR (qPCR)

Porcine valvular interstitial cells were harvested, and total RNA was extracted from cells using TRIzol reagent (Bioteke Beijing, China) and then reverse-transcribed using a first-strand cDNA synthesis kit (TSK302S, RT6 cDNA Synthesis Kit Ver 2). Reverse-transcribed products were used as templates for qPCR using 2 × T5 Fast qPCR Mix (SYBR Green I). The primers used for different target genes (CAT’s different UTR regions) were as follows: Short-APA-F: 5′-TCCAGTGACGAATGGGTATG-3′; Short-APA-R: 5′-TCTGCCTCTGAAGCAAAACA-3′; Long-APA-F: 5′-CTCATCAGTTCCAAGCAGGA-3′; Long-APA-R: 5′-AATGCTTTGGGGTCTTTTGA-3′. BGLAP-F: 5′-CGCTACCTGTATCAATGGCTGG-3′; BGLAP-R: 5′-CTCCTGAAAGCCGATGTGGTCA-3′. RUNX2-F: 5′- CCCAGTATGAGAGTAGGTGTCC-3′; RUNX2-R: 5′-GGGTAAGACTGGTCATAGGACC-3′; IBSP-F: 5′-GGCAGTAGTGACTCATCCGAAG-3′; IBSP-R: 5′ -GAAAGTGTGGTATTCTCAGCCTC-3′. and beta-actin was used as the control. Actin-F: 5′-CCCTGGAGAAGAGCTACGAG-3′; Actin-R: 5′-GTGATCTCCTTCTGCATGCG-3′. The qPCR thermal cycle parameters (40x) are: 95 °C 20s, 60 °C for 20s, 72 for 20s.

### Western blotting

Both bFGF and OIM-treated cells were collected for protein extraction. The cells were washed three times before preparation. Total lysates were separated using sodium dodecyl sulfate-polyacrylamide gel electrophoresis (SDS-PAGE) and transferred to nitrocellulose (NC) membranes for 1 h. The membranes were then blocked for 45 min with Tris-buffered saline containing Tween 20 (TBST) and 3% (mass/vol) nonfat dry milk. After incubation with primary antibodies (1:1000), the membranes were washed and incubated with HRP-conjugated anti-mouse or anti-rabbit secondary antibodies (1:10000). The membrane was visualized using enhanced chemiluminescence (ECL) western blotting substrate (Bio-Rad, Hercules, CA, USA).

Antibody catalog numbers are: Osteopontin (OPN) (ab69498, 1:1000) from Abcam; Runt-related transcription factor 2 (RUNX2) (20700–1-AP, 1:1000) from Proteintech; CAT (ab76110, 1:1000) from Abcam; Actin (ab6276, 1:5000) from Abcam.

### Statistical analysis

All results are presented as mean ± standard deviation. Student’s t-test was used to analyze the statistical differences between the two groups. All statistical tests were executed using GraphPad Prism 8.0.2 and SPSS 22.0. Differences with *p* < 0.05 were considered statistically significant.

## Results

### OIM induced calcium deposition and upregulated OPN and RUNX2

**Fig. 1:**
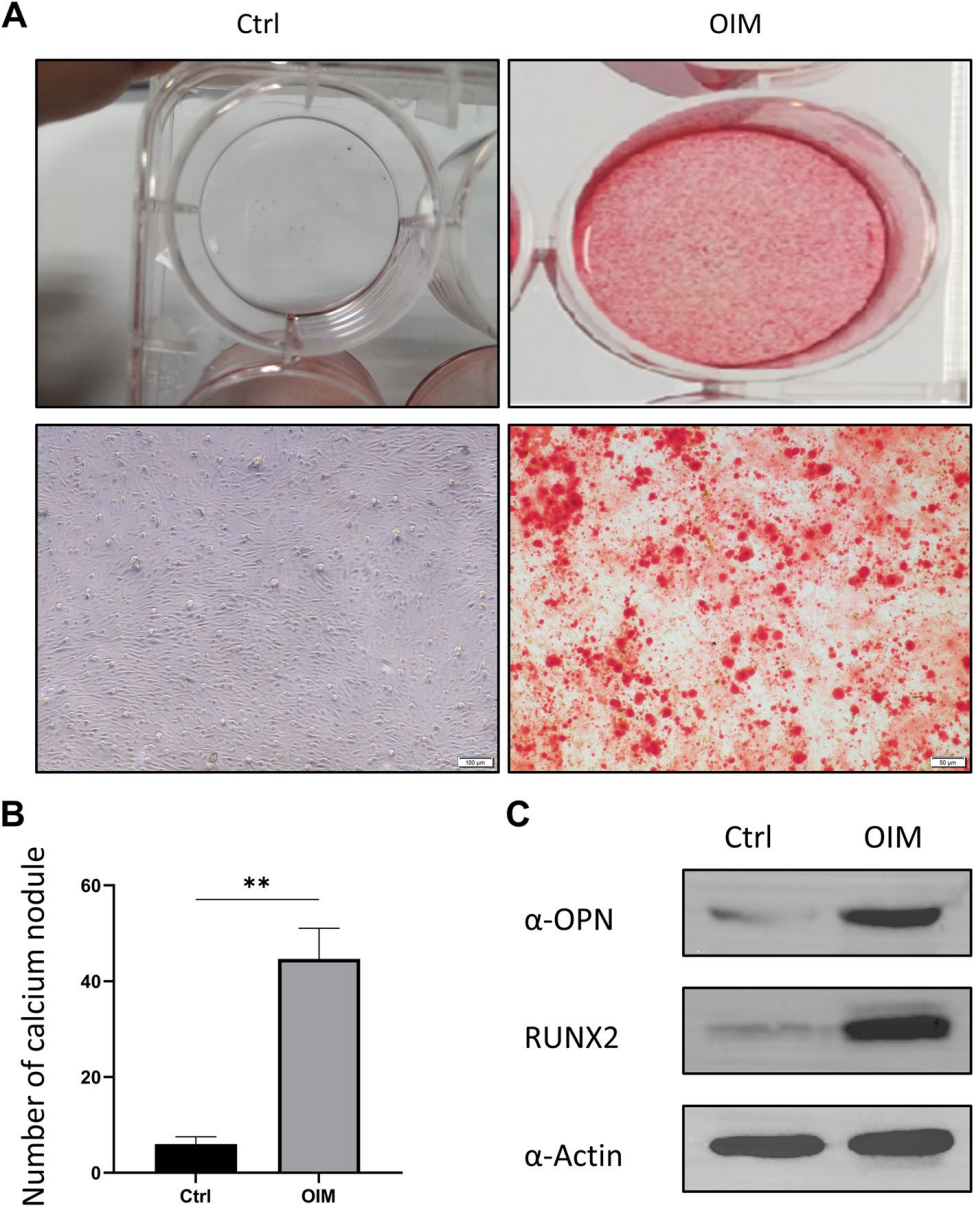
Alizarin Red S staining and western blotting for OPN of PVICs induced by OIM. **A**, Alizarin Red S staining of PVICs induced by osteogenic medium OM for 7 days, control (normal culture medium), OIM (osteogenic medium). B, Quantitative analysis of calcium nodules before and after OIM treatment. **C**, The protein expression of OPN and RUNX2 were increased induced by OIM for 72 hrs. Actin was used as the control. OIM = osteogenic medium

OIM-induced osteogenic differentiation was performed on PVICs for 7 days. The OIM-treated PVICs were stained red (positive for Alizarin Red), indicating nodule formation, as compared to the control group (Fig. 1A-B). The osteogenesis-specific protein OPN and RUNX2 were tested after culturing the PVICs in each group for 3 days. Western blotting demonstrated that OIM significantly upregulated the protein expression of OPN and RUNX2 compared to that in the control group (Fig. 1C). These results suggest that OIM-induced calcium deposition was successful.

### mRNA-seq identifies differentially expressed mRNAs among three different treatments

**Fig. 2:**
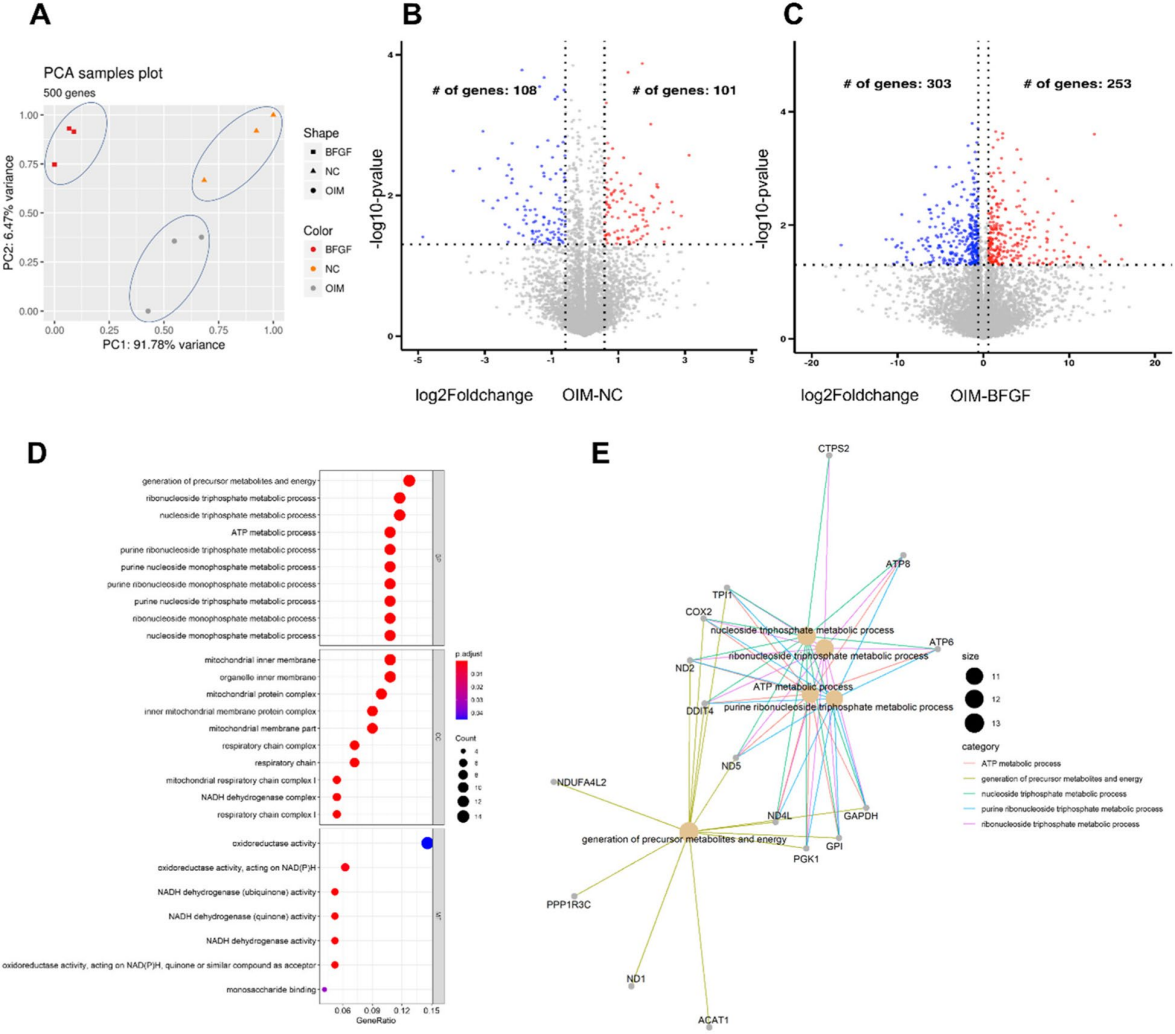
mRNA-seq identifies differentially expressed mRNAs among three different treatments. **A**, PCA plot shows all 9 samples from three treatment were well separated. NC: Negative Control. **B,C**, Volcano plot shows differentially expressed genes of OIM vs control and OIM vs bFGF-treated, respectively. **D**, Dotplot shows the significantly enriched pathways in differentially expressed genes in bFGF treated group. **E**, CNEplot shows the interaction of these enriched GO terms. OIM = osteogenic medium

As PVICs undergo calcium deposition under OIM treatment, this may be reflected as the formation of CAVD in vivo. We performed mRNA-seq on bFGF- and OIM-treated PVICs to reveal transcriptome changes in treated PVICs compared with untreated cells. The experiments were divided into three groups according to the different treatments that PVIC received: control, OIM-induced and OIM-induced bFGF treatment (see Methods). Each group was sequenced in triplicate. We generated a high-quality RNA-seq dataset in terms of sequencing quality, duplication removal, adapter contamination, and mapping ratios. The Principal Component Analysis (PCA) plot demonstrated that the transcriptomic profiles of PVICs subjected to different treatments were well separated (Fig. 2A). Differential expression analysis between the OIM and control group using DEseq2 revealed that 101 and 108 genes were significantly upregulated and downregulated, respectively (Fig. 2B).

We observed a significant upregulation of genes associated with calcium deposition and inflammatory processes, including *SIPA1L1*, *SUN2*, *MVP*, *TOMM40*, *LPP*, and *ATF4*. This indicates the extensive inflammatory and calcium deposition processes occurring in endothelial cells following OIM stimulation. Compared to the bFGF-treated group, 253 and 303 genes were significantly upregulated and downregulated, respectively in the OIM group (Fig. 2C). After treatment with bFGF, we observed a transcriptional enhancement of anti-inflammatory processes as evidenced by the upregulation of the transcription of *SPP1*, *ITGA2*, *S100A2*, *APP*, and *ZEB2*, as well as the inhibition of calcium deposition processes. These results indicated that both OIM and OIM-bFGF treatments could alter the transcriptome profiles of PVIC cells. Next, we examined the involvement of bFGF-related signals in OIM-induced calcium deposition, as our original hypothesis was that bFGF treatment can inhibit the OIM-induced calcium deposition process. We examined the enriched pathways in the bFGF-treated group compared to the OIM group using the GO enrichment analysis. We found that energy and nucleotide metabolism pathways, such as ‘generation of precursor metabolites and energy’, ‘ribonucleoside triphosphate metabolic process’, were significantly enriched in the bFGF group (Fig. 2D-E), which matched previous conclusions regarding the impact of bFGF on energy metabolism [15], cell proliferation, and calcium deposition [16]. These results support previous findings on bFGF-related signaling pathways.

### Global APA events were identified in the OIM and bFGF-treated groups

**Fig. 3:**
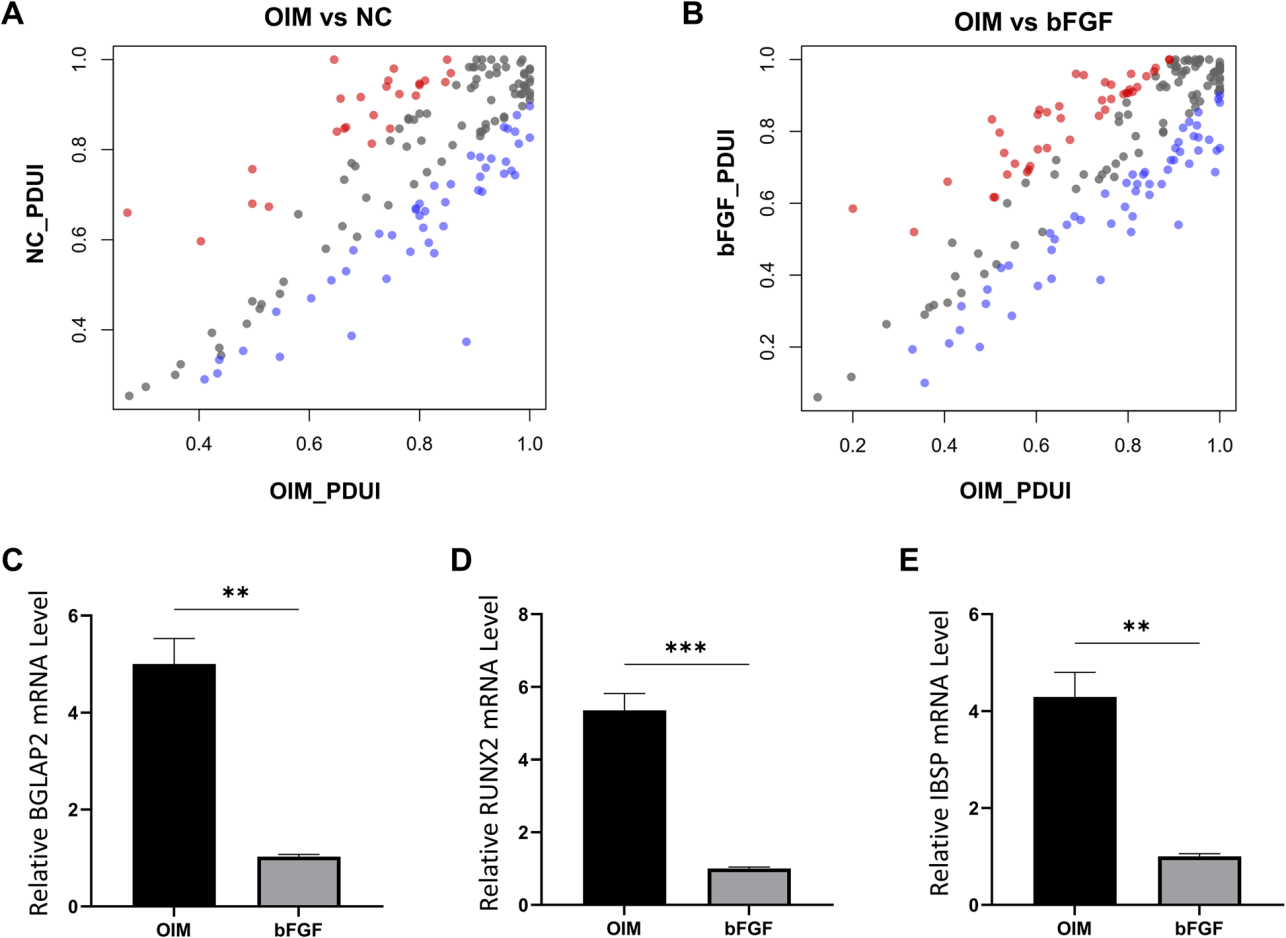
Global APA events were identified in OIM and bFGF treated groups. **A,B**, Scatter plot showed the significant dynamic APA events in OIM vs Ctrl and OIM vs bFGF treatment. Red dots refer to lengthened genes; Blue dots refer to shortened genes. C,D,E, The expression of BGLAP2, RUNX2 and IBSP was detected by qPCR before and after bFGF treatment. APA events when PDUI_diff cutoff is set as 0.1. APA = alternative polyadenylation, OIM = osteogenic medium, PDUI = distal polyA site usage index

**Fig. 4:**
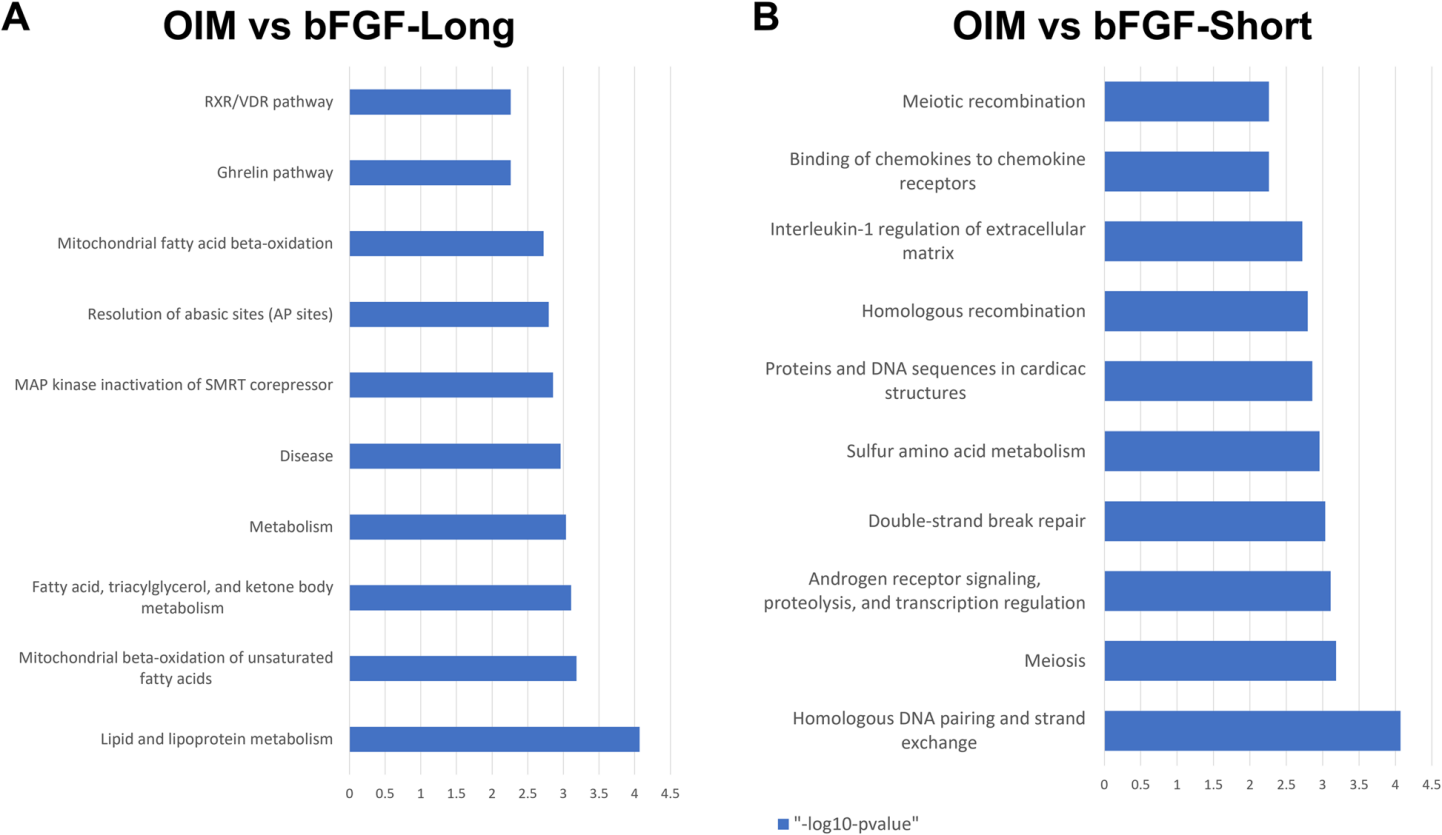
GO analysis of bFGF induced significant APA genes. **A,B,** Lengthened and shortened APA genes’ GO enrichment analysis. BioPlanet 2019 was used as the GO dataset. APA = alternative polyadenylation

We were interested in evaluating whether OIM-induced calcium deposition and bFGF treatment were associated with APA events, thus contributing to the change in their transcriptomic profiles. We successfully identified significant APA differences between the OIM and control groups and the bFGF and OIM groups using DaPars software. The results indicate that APA may play an important role in regulating bFGF- and OIM-induced cellular signals (Fig. 3A-B). In addition to analyzing global APA, we evaluated the expression of osteogenic genes *BGLAP*, *RUNX2*, and *IBSP*. Our results revealed that under bFGF treatment, the expression levels of *BGLAP* and *RUNX2* were notably reduced compared to those in the OIM group (Fig. 3C-E). Interestingly, in the bFGF group, shorter 3′-UTRs were more frequent than the lengthened events (1.4-fold higher), which also supported the previous conclusion that EGF-related signals could shorten the 3′-UTR [10]. We were also interested in whether these bFGF-related significant APA events were associated with certain cellular reactions. Therefore, we performed GO analysis (bioPlanet database) on both lengthened and shortened APA genes. We found that “lipid and lipoprotein metabolism”, fatty-acid related pathways were significantly enriched in the lengthened APA events (Fig. 4A), while “homologous DNA pairing and strand exchange”, “double-strand break repair, and cardiac structure-related pathways were profoundly enriched in the shortened APA events (Fig. 4B). Interestingly, DNA repair and oxidative stress were previously associated with bFGF signals [17, 18], as bFGF has been recognized as a driver of DNA repair. Our results, as well as those of these cancer studies, suggest that treatment with bFGF and OIM may affect DNA repair and oxidative stress by regulating APA events, thus contributing to the etiology of CAVD.

### *CAT* is one of the potential targets under the regulation of bFGF-induced APA

**Fig. 5:**
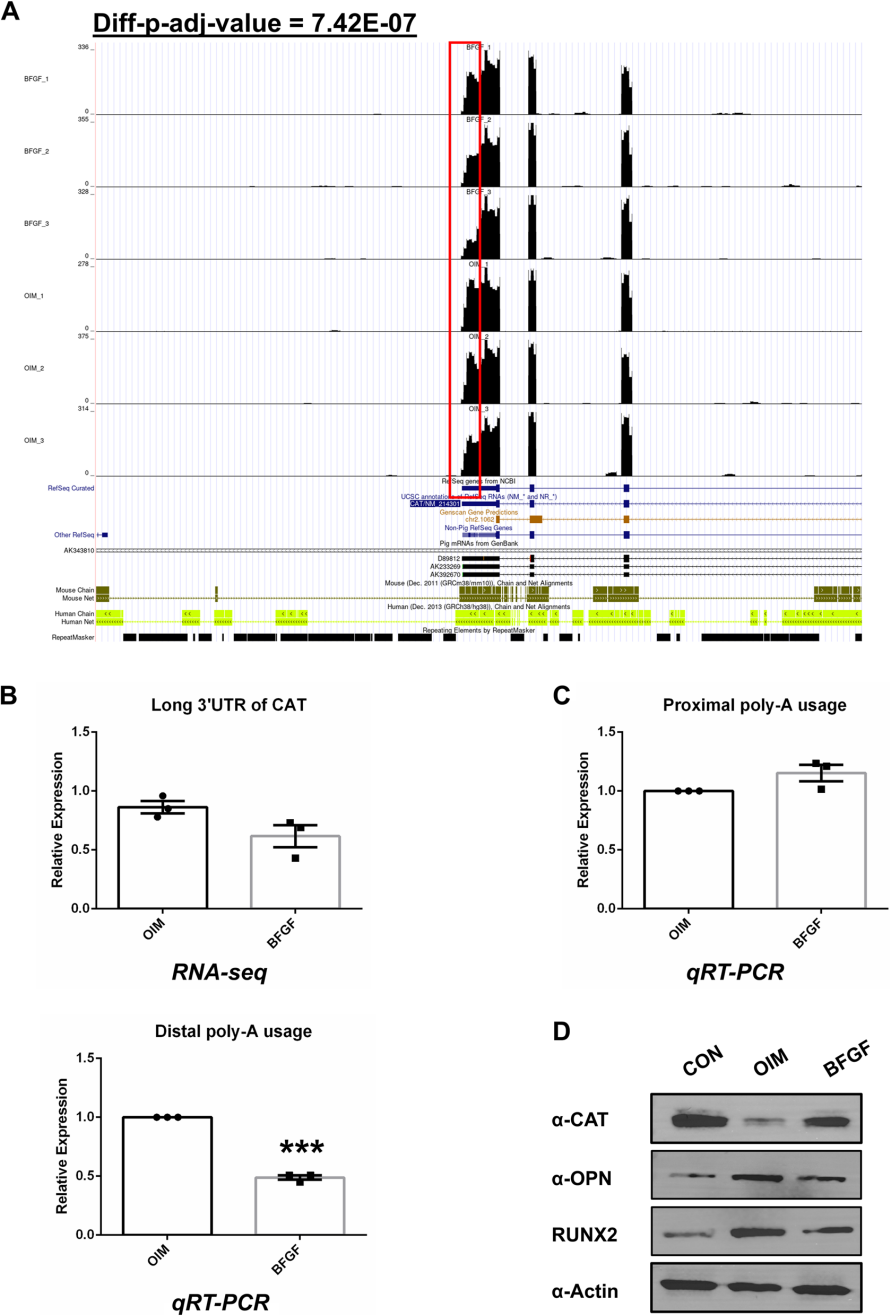
Catalase is one of the potential targets under the regulation of bFGF induced APA. **A,** UCSC genome browser track figure demonstrates all three samples under bFGF treatment has shortened APA event in *CAT* gene. **B,** RNA-seq and qPCR results of the distal APA expression level. ***: *p* < 0.001 **C,** qPCR results showed the proximal poly-A usage of *CAT* gene is not significantly altered. **D**, Western blot assays showed the protein expression of CAT is increased and OPN and RUNX2 are decreased in bFGF group. APA = alternative polyadenylation, CAT = catalase, OPN = Osteopontin, RUNX2 = Runt-related transcription factor-2

As bFGF signals were highly associated with oxidative stress, we examined whether any of the significantly affected APA genes were involved in the related pathways. We noticed that CAT gene UTR usage was significantly altered in all bFGF-treated cell samples (Fig. 5A). Statistical analysis based on DaPars demonstrated that distal poly-A usage was significantly decreased in the bFGF-treated group (p-adj-value = 7.42E-07, FDR adjusted), indicating a bFGF-specific shortening of the APA of *CAT*. We performed qRT-PCR to validate this finding. The expression of the distal poly-A region was significantly downregulated in the bFGF group (Fig. 5B, p < 0.001), while proximal poly-A usage was not significantly altered (Fig. 5C), which supported the RNA-seq findings (Fig. 5A). Finally, we were interested in whether APA in *CAT* also affected the protein level of CAT. We performed western blot assays on bFGF-OIM-treated cells, OIM-treated cells and control cells to detect CAT protein levels. We found that the CAT protein level was increased, along with the decreased protein level of OPN and RUNX2 in the bFGF treated cells (Fig. 5D), indicating that APA event of the *CAT* gene affected its protein translation; thus, it may act as a potential target of bFGF induced inhibition of calcium deposition by inhibiting oxidative stress [19].

## Discussion

Despite years of effort devoted to CAVD research, no medical therapies for CAVD have proven effective in halting CAVD progression as an alternative to surgery. Techniques based on next-generation sequencing have the potential to identify the regulatory mechanisms of CAVD. In this study, we utilized an OIM to simulate CAVD in PVICs, focusing on calcium deposition. Our findings indicated that OIM successfully induced calcium deposition, as evidenced by specific staining and upregulation of osteogenic markers. Intriguingly, treatment with bFGF demonstrated a mitigating effect on this calcium deposition, suggesting its therapeutic potential. Through mRNA sequencing, we observed significant transcriptomic changes in PVICs under different treatments. Crucially, our study revealed the role of APA in these cellular responses, particularly under bFGF treatment, which affected mRNA processing and gene expression, notably in the CAT gene. This finding was further supported by changes at the protein level. Collectively, these results highlight the complex interactions between bFGF treatment, gene regulation through APA, and their implications in CAVD, underscoring the potential of bFGF as a modulator of disease progression.

Our study indicates a significant enrichment of energy and nucleotide metabolism pathways in the bFGF-treated group, corroborating previous research findings. For instance, bFGF treatment has notably reduced lipid levels, including low-density and very low-density lipoproteins, as well as glucose in the serum of diabetic rats [15]. This result underscores the substantial influence of bFGF on energy metabolism by modifying a range of related metabolites. Moreover, another study examining bFGF’s protective effect against diabetic nephropathy in mice has illuminated its role in reshaping metabolic phenotypes and attenuating oxidative stress. These studies collectively affirm bFGF’s capability to modulate metabolic pathways, providing additional evidence of its regulatory role in metabolism. The potential impact of bFGF on calcium deposition is also suggested, further expanding the scope of its biological significance.

bFGF and EGF signals work orchestrally on cell proliferation, osteogenesis, and other basic cellular processes [8]. Recently, EGF signals were found to be highly associated with alternative splicing and APA processes in cancers and other developmental diseases [10], especially for their role in 3’UTR shortening. APA is a crucial post-transcriptional regulatory mechanism that is involved in drug sensitivity and disease progression [6]. With our substantially enriched knowledge of APA, this widespread phenomenon may be a potential biomarker for disease prognosis and diagnosis, such as CAVD, and a target for the development of novel targeted therapies [20, 21]. Our results demonstrate, for the first time, the APA changes in bFGF- and OIM-treated PVICs. We found that bFGF and OIM may affect DNA repair and oxidative stress in PVICs by regulating APA events, thus contributing to the etiology of CAVD. Our data suggest that bFGF- and OIM-related APA events may be effective targets in novel targeted therapies for CAVD and a promising biomarker for CAVD prognosis and diagnosis.

CAT is a crucial enzyme that protects cells from oxidative damage by reactive oxygen species (ROS) in nearly all living organisms exposed to oxygen [22]. Overexpression of CAT in vivo protects the process of vascular calcium deposition [23]. CAT is downregulated in the calcified region of human aortic valves, and the introduction of CAT reverted ROS-mediated calcium deposition in VICs [24]. Based on previous studies on the relationship between CAT and calcium deposition, we hypothesized that manipulating *CAT* in VICs may be a useful strategy for treating CAVD. We observed a significant decrease in *CAT* gene UTR usage in all bFGF-treated cell samples, indicating a bFGF-specific shortening event of the *CAT* gene APA. Our findings suggest that bFGF might rescue calcium deposition in PVICs by shortening the APA length of *CAT*, thus reducing oxidative stress-mediated calcium deposition in PVICs, to revert the effect of CAVD.

Our findings provide insights into how bFGF may function in PVICs. We connected APA events to CAVD and investigated the APA change of a specific gene, *CAT*, which appears to be an important gene in ROS-mediated calcium deposition regulation. We propose that careful manipulation of APA in CAT may be useful in rescuing calcium deposition in PVICs, thus treating CAVD.

### Supplementary Information

**Supplementary material 1.**

## Data Availability

The data is deposited at NCBI GEO dataset with accession number GSE225698. The temporary code is kvezqooufdurpgh for accessing the GEO dataset.
