# Supplementary material for: Inhibition of valve mesenchymal stromal cell calcium deposition by bFGF through alternative polyadenylation regulation of the CAT gene
Source: BMC Cardiovasc Disord. 2024 Feb 28;24:128. doi: 10.1186/s12872-024-03775-5 (PMC10903013; doi:10.1186/s12872-024-03775-5)
Supplement: Supplementary file 1 — Supplementary material 1. [file 12872_2024_3775_MOESM1_ESM.zip › BFGF WB raw data-submission.pptx]

## Slide 1
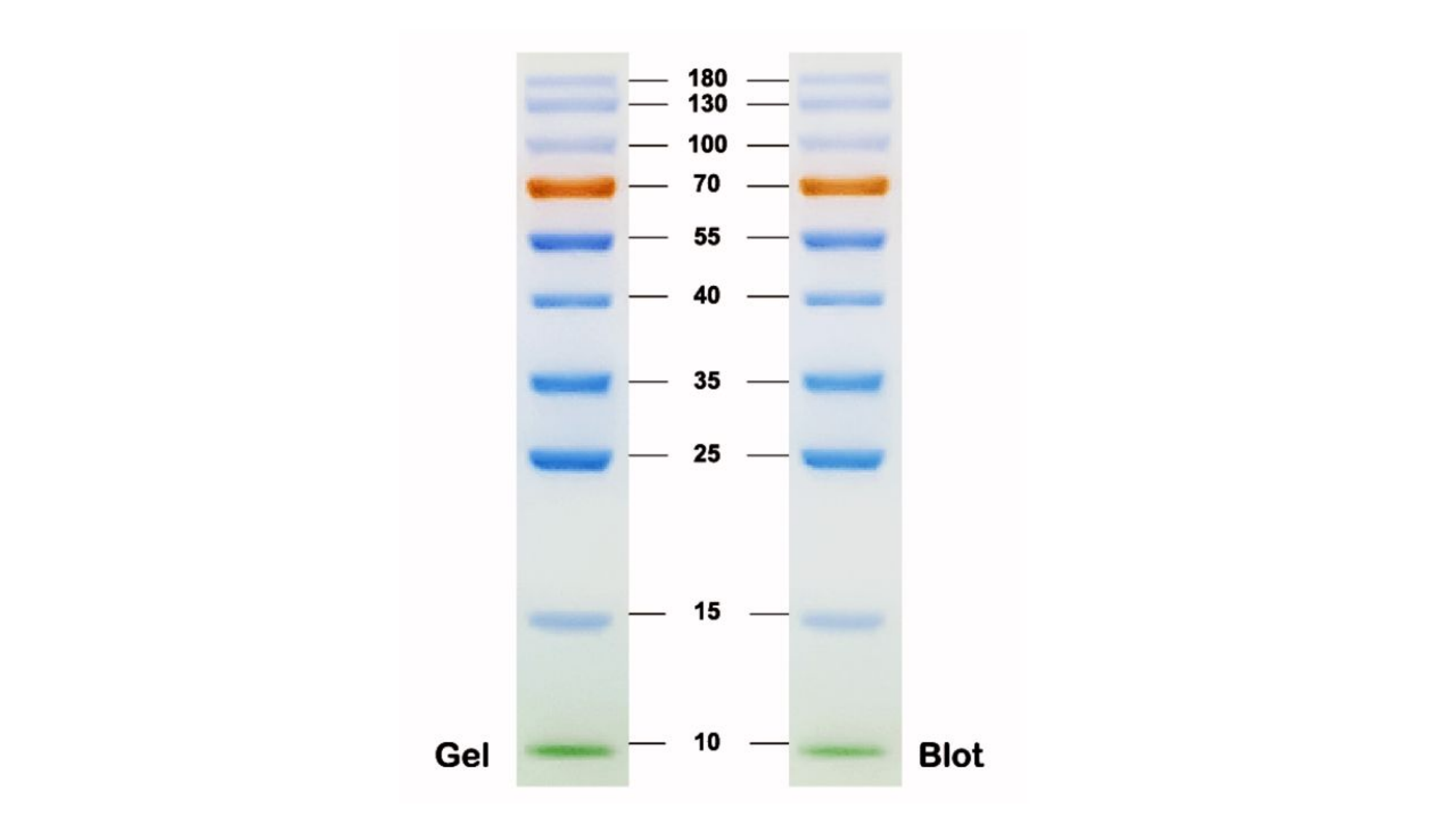

## Slide 2
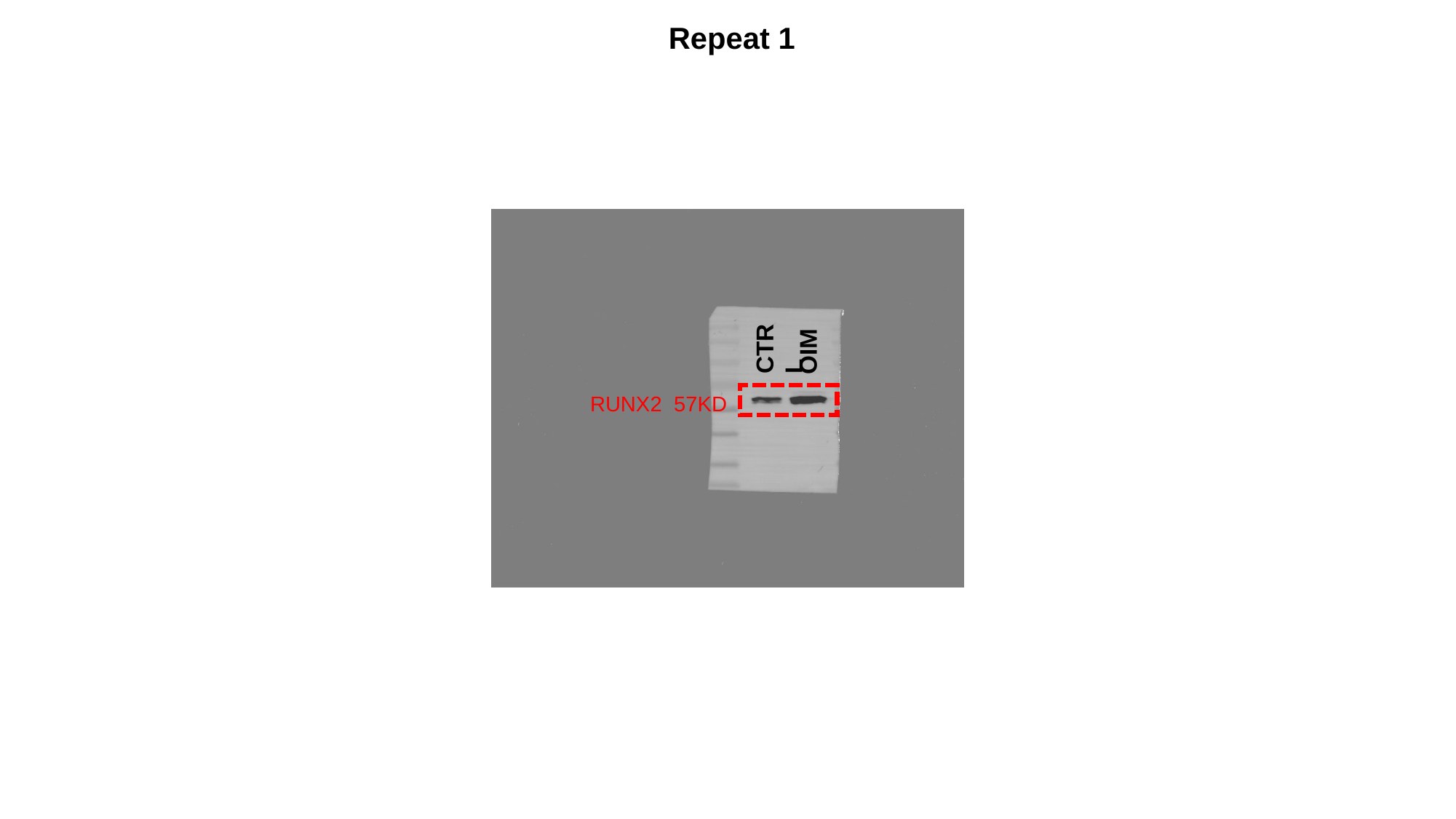

Repeat 1
OIM
CTRL
RUNX2 57KD

## Slide 3
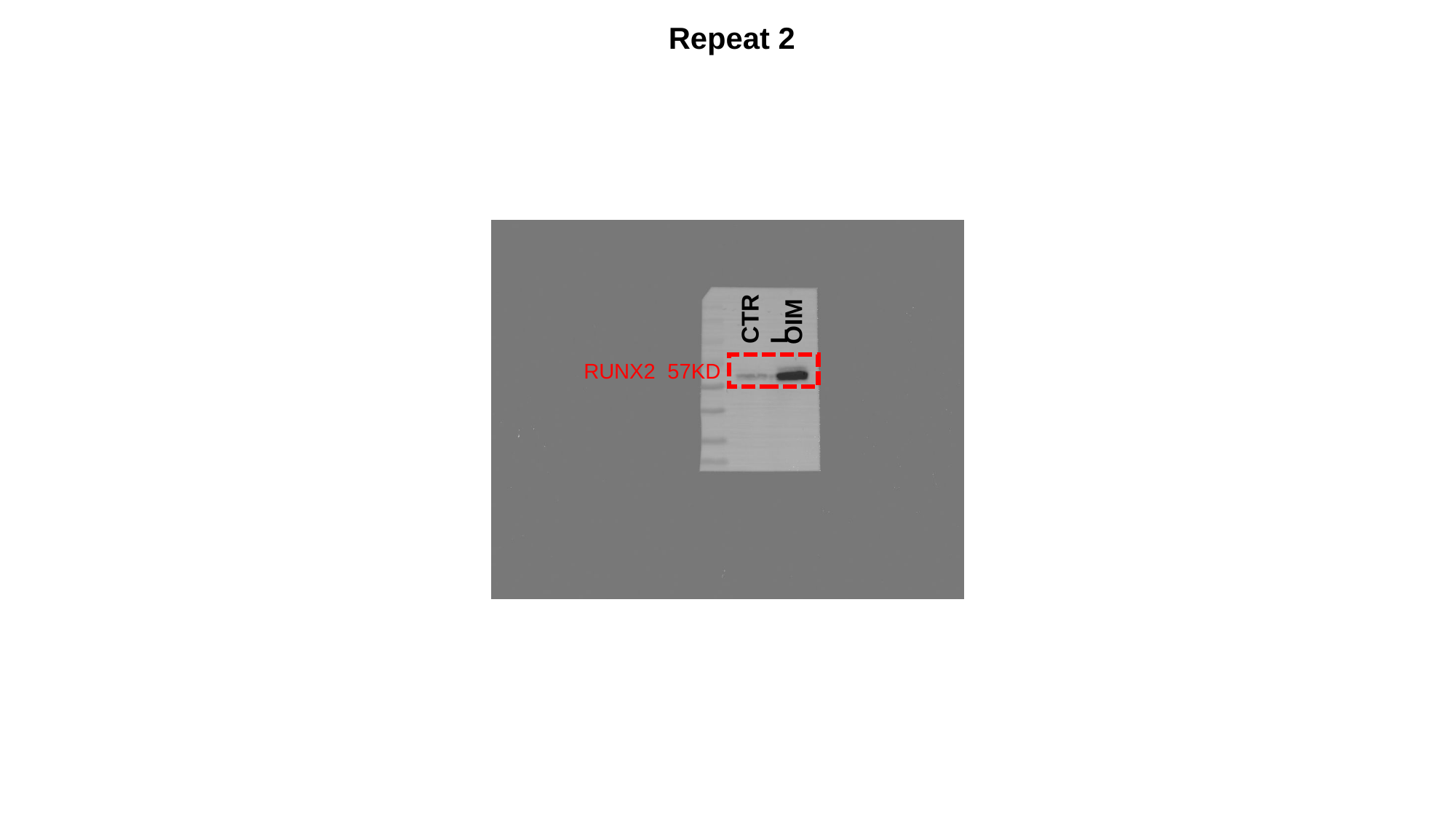

Repeat 2
OIM
CTRL
RUNX2 57KD

## Slide 4
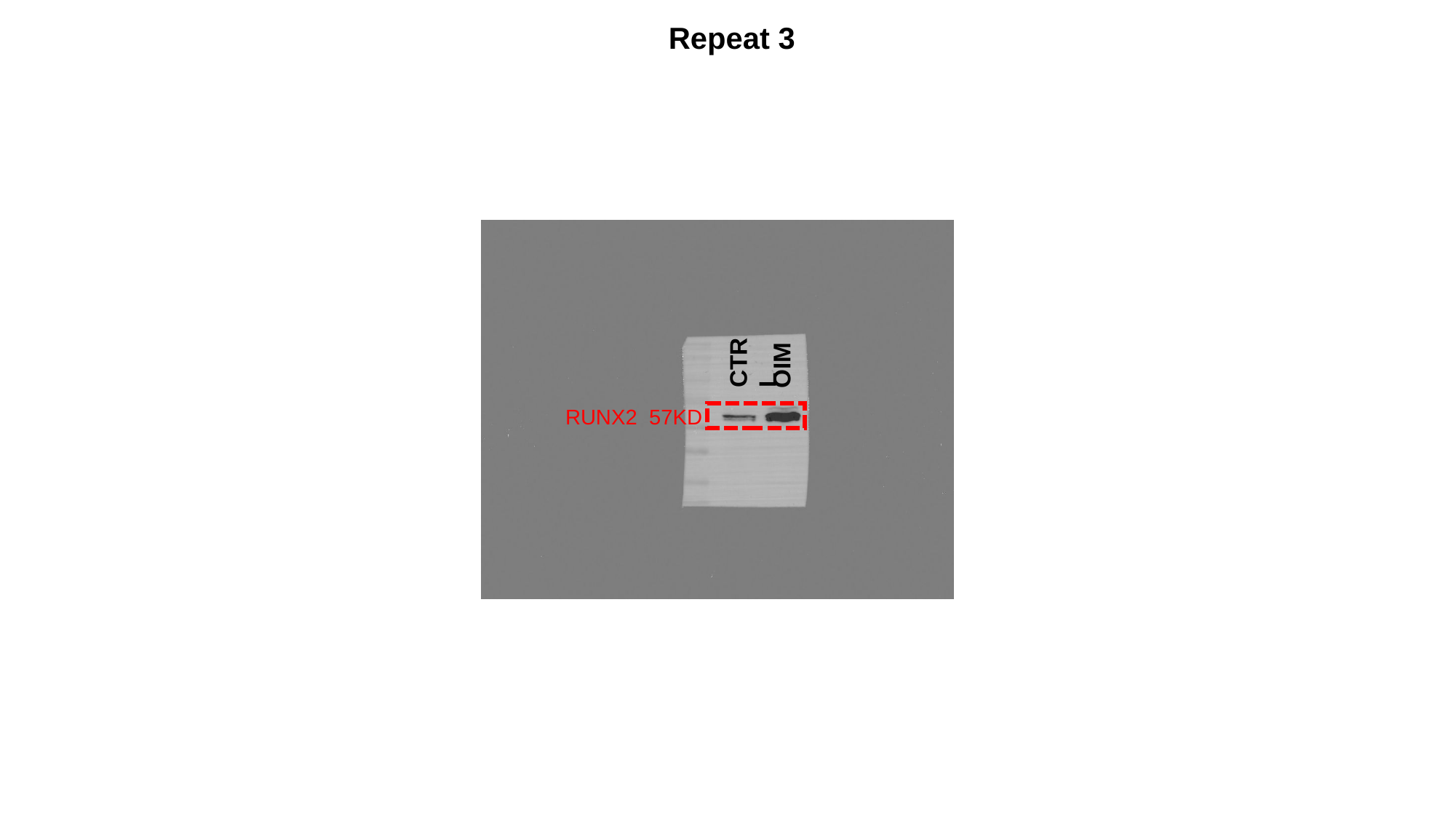

Repeat 3
OIM
CTRL
RUNX2 57KD

## Slide 5
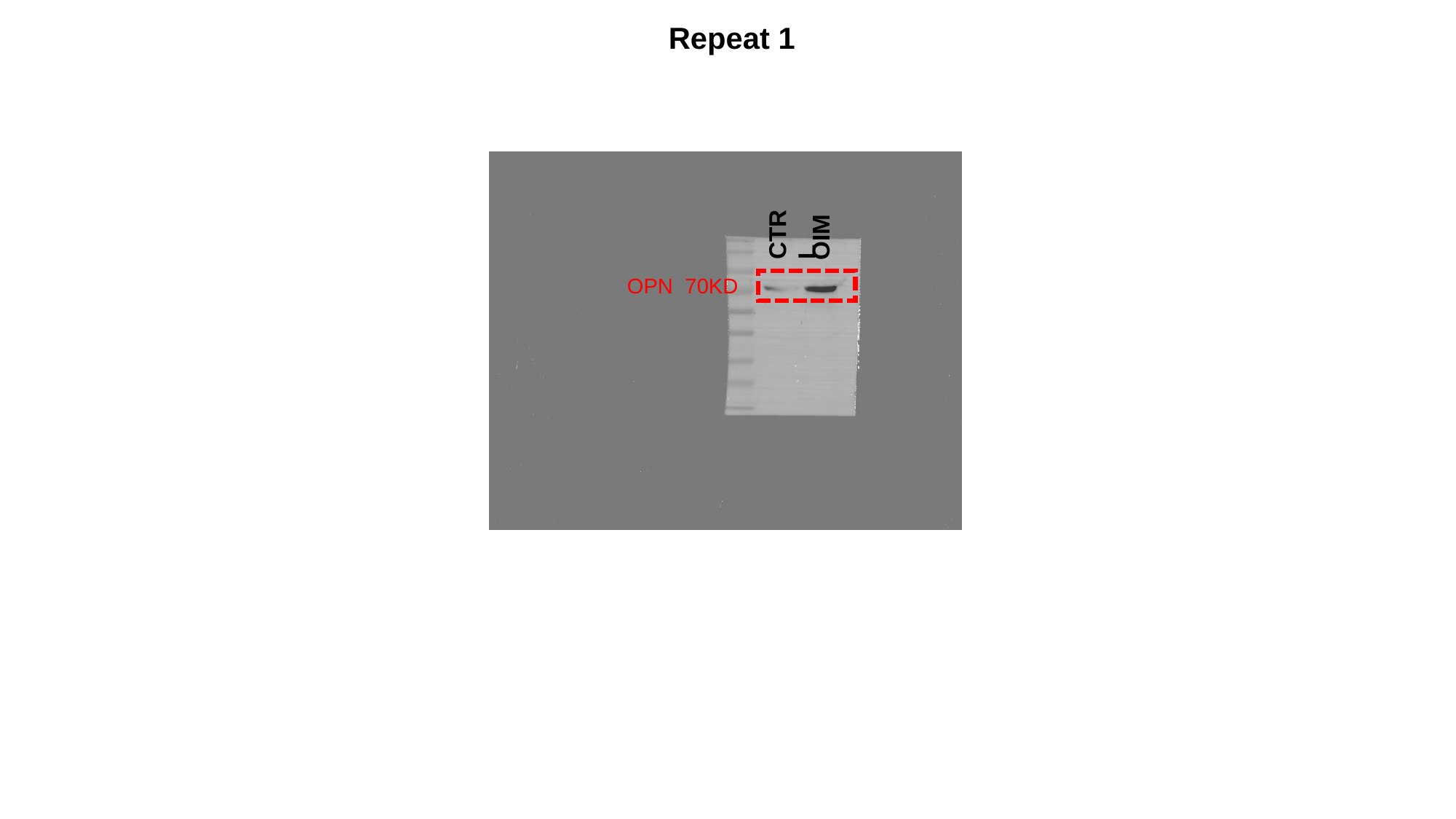

Repeat 1
OIM
CTRL
OPN 70KD

## Slide 6
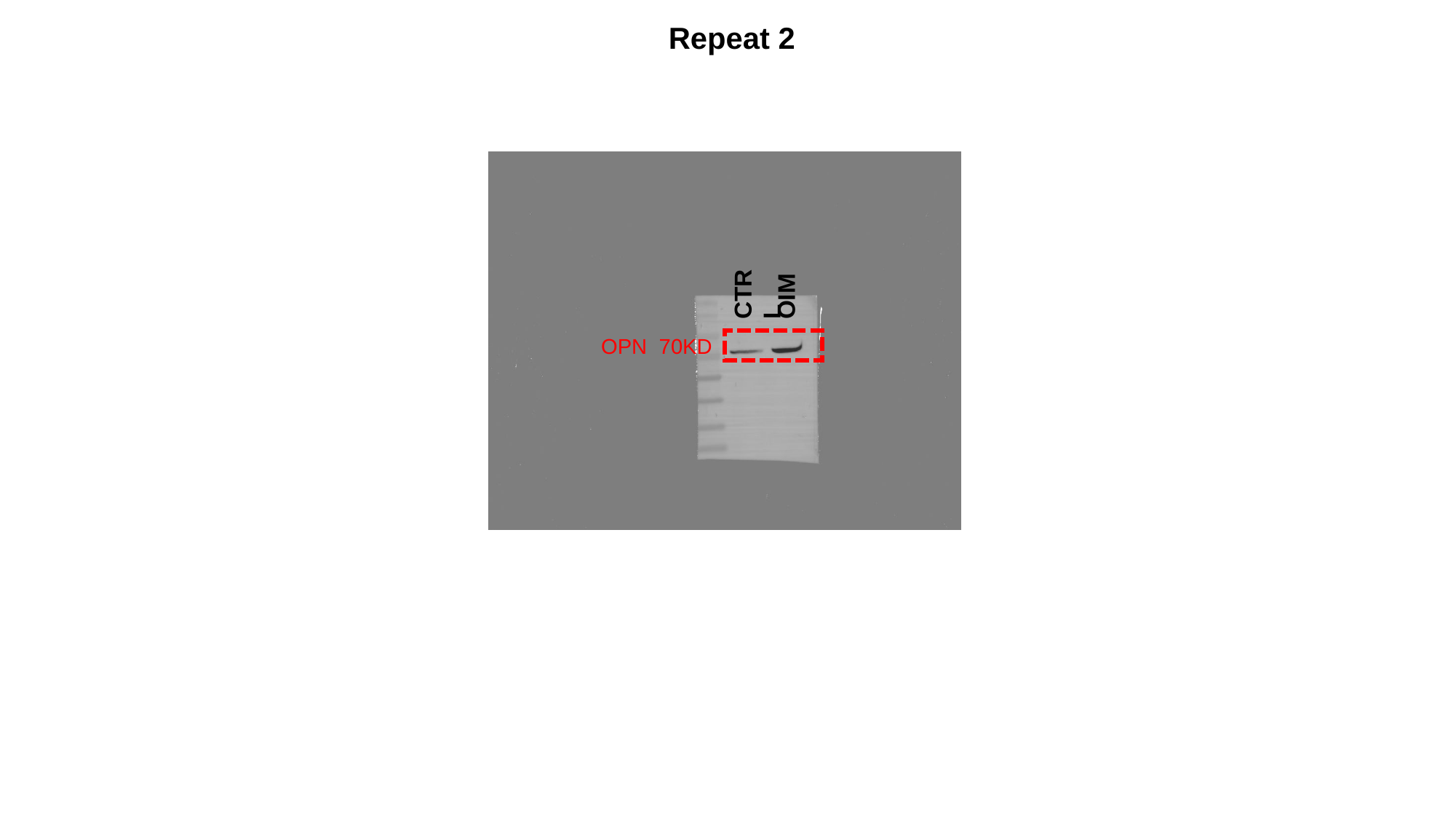

Repeat 2
OIM
CTRL
OPN 70KD

## Slide 7
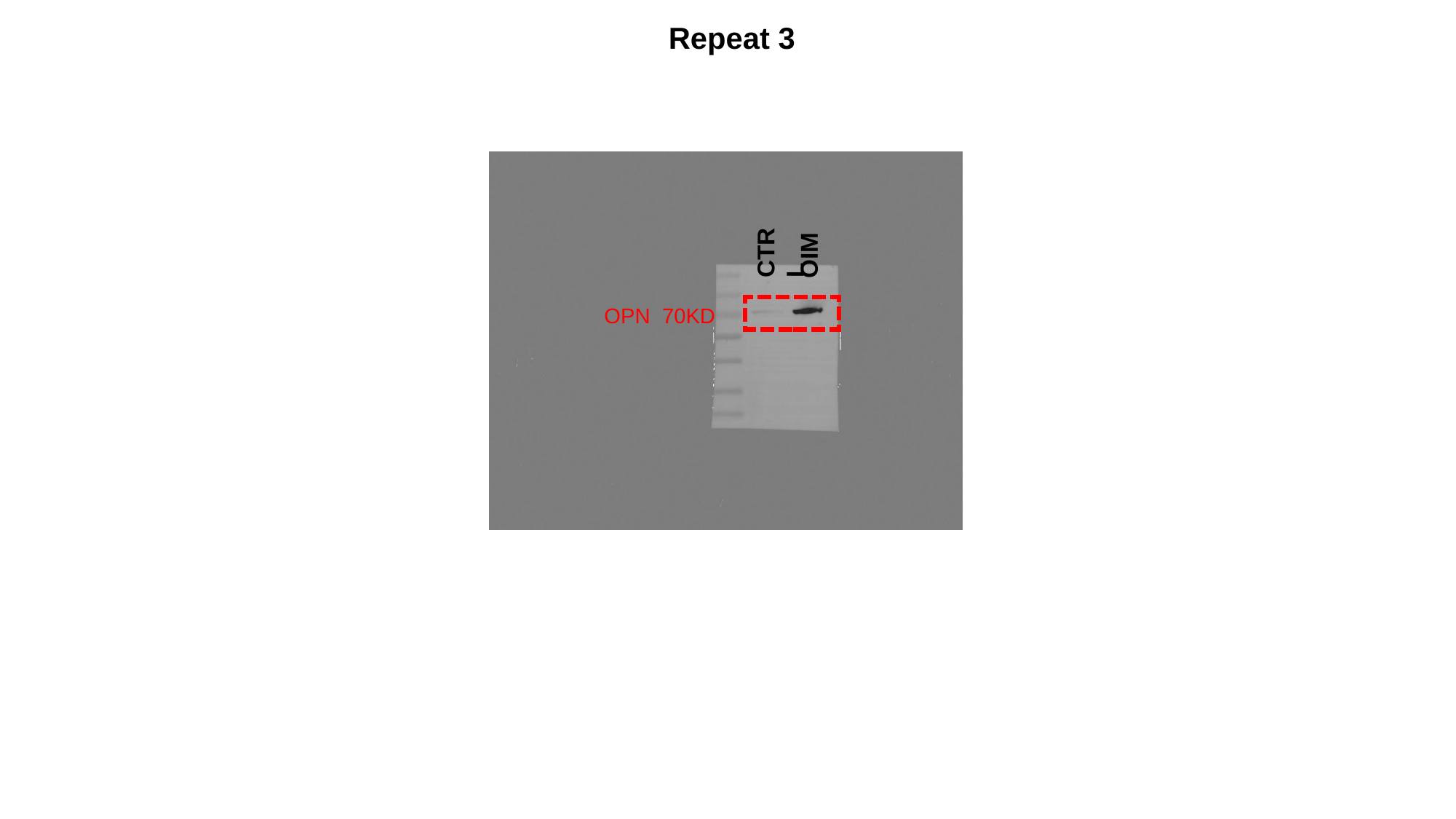

Repeat 3
OIM
CTRL
OPN 70KD

## Slide 8
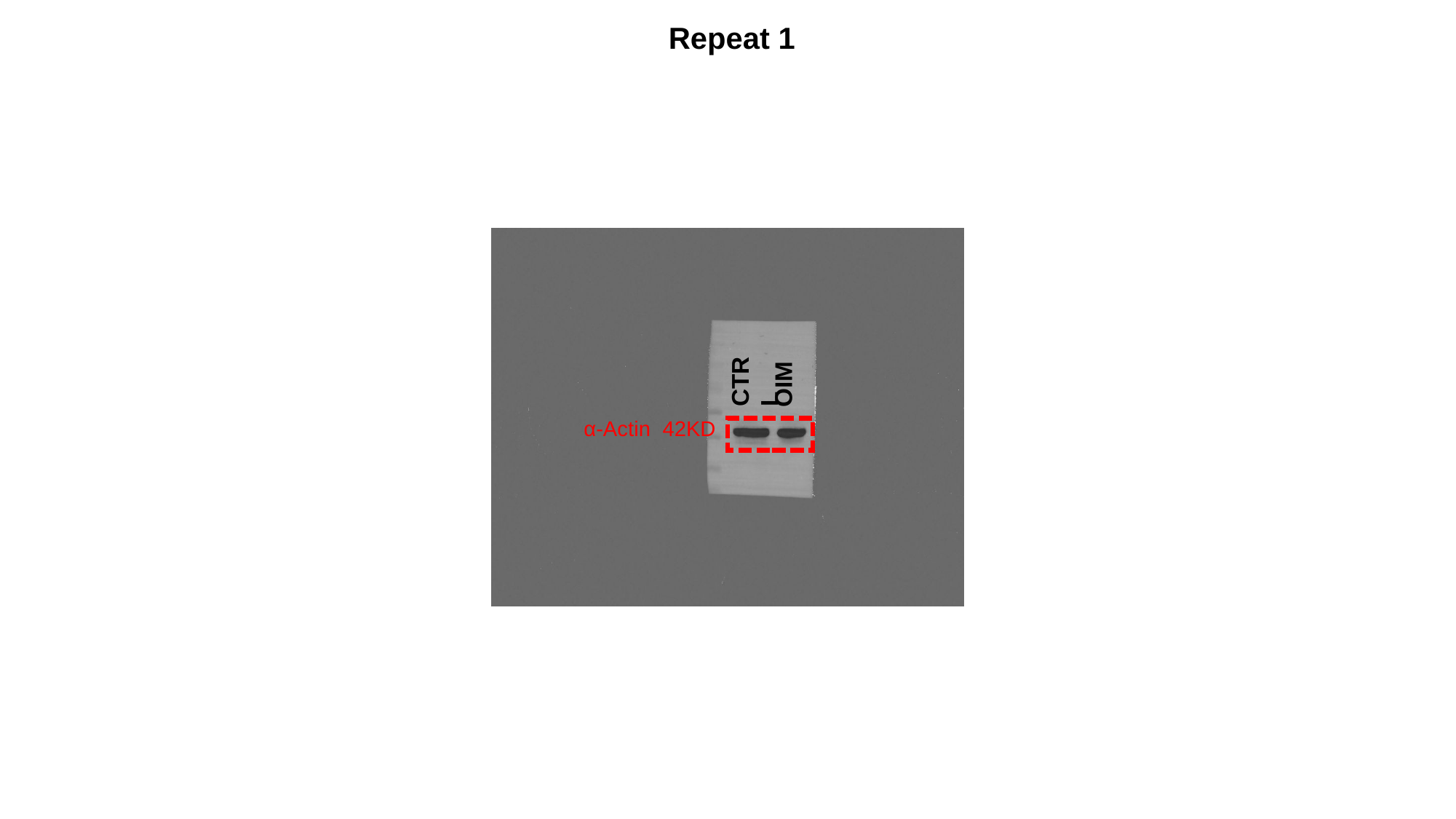

Repeat 1
OIM
CTRL
α-Actin 42KD

## Slide 9
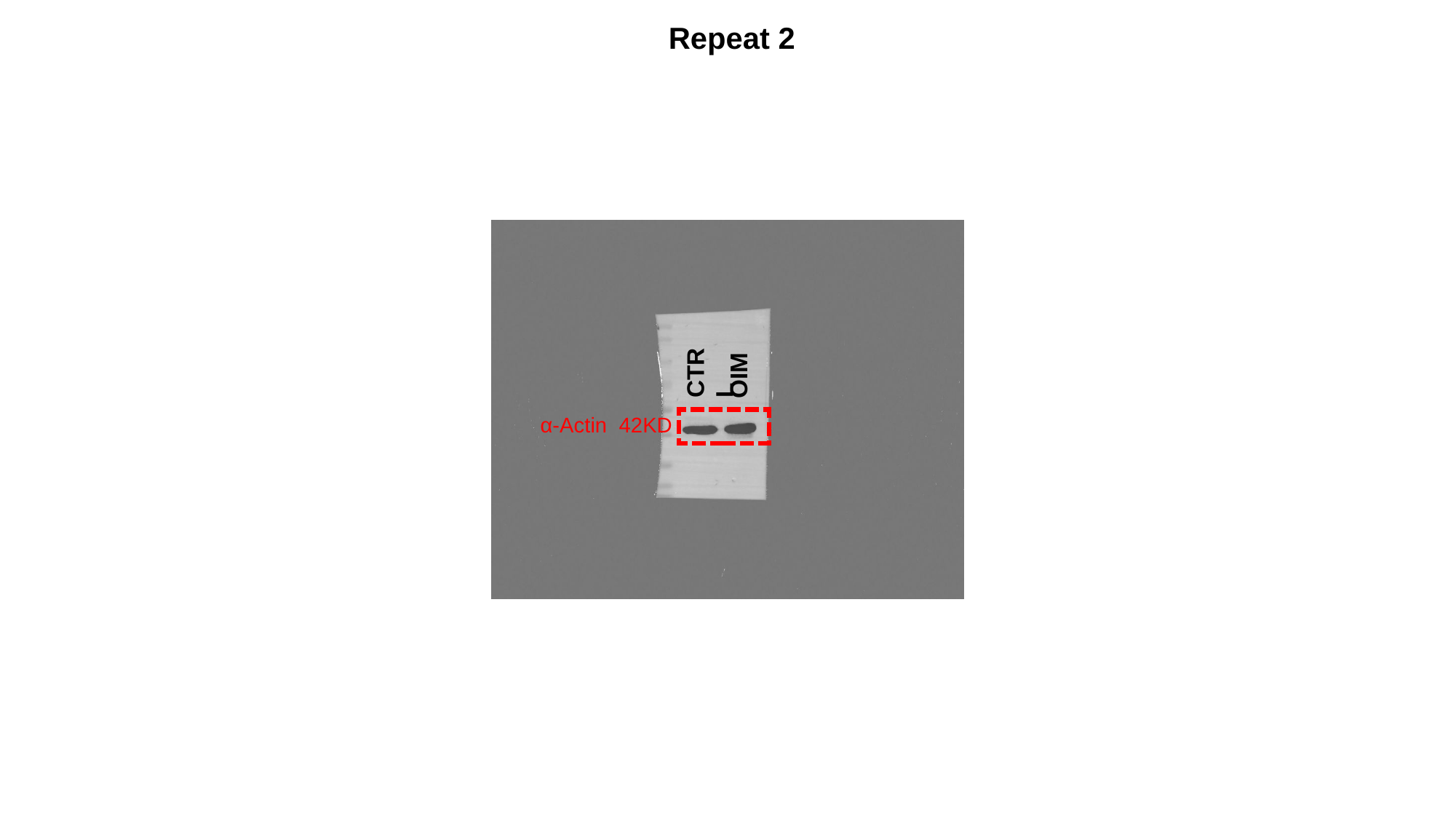

Repeat 2
OIM
CTRL
α-Actin 42KD

## Slide 10
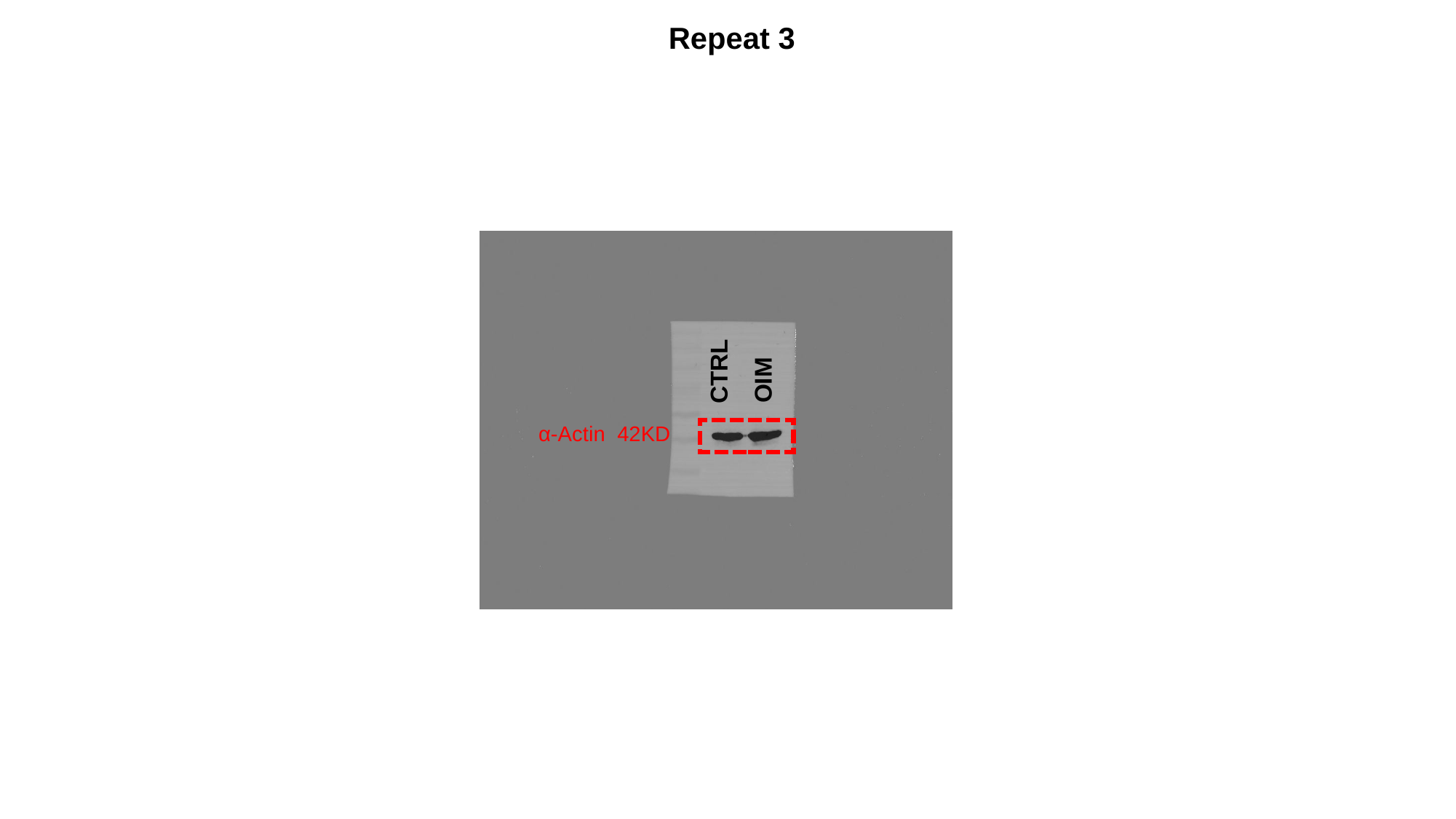

Repeat 3
OIM
CTRL
α-Actin 42KD

## Slide 11
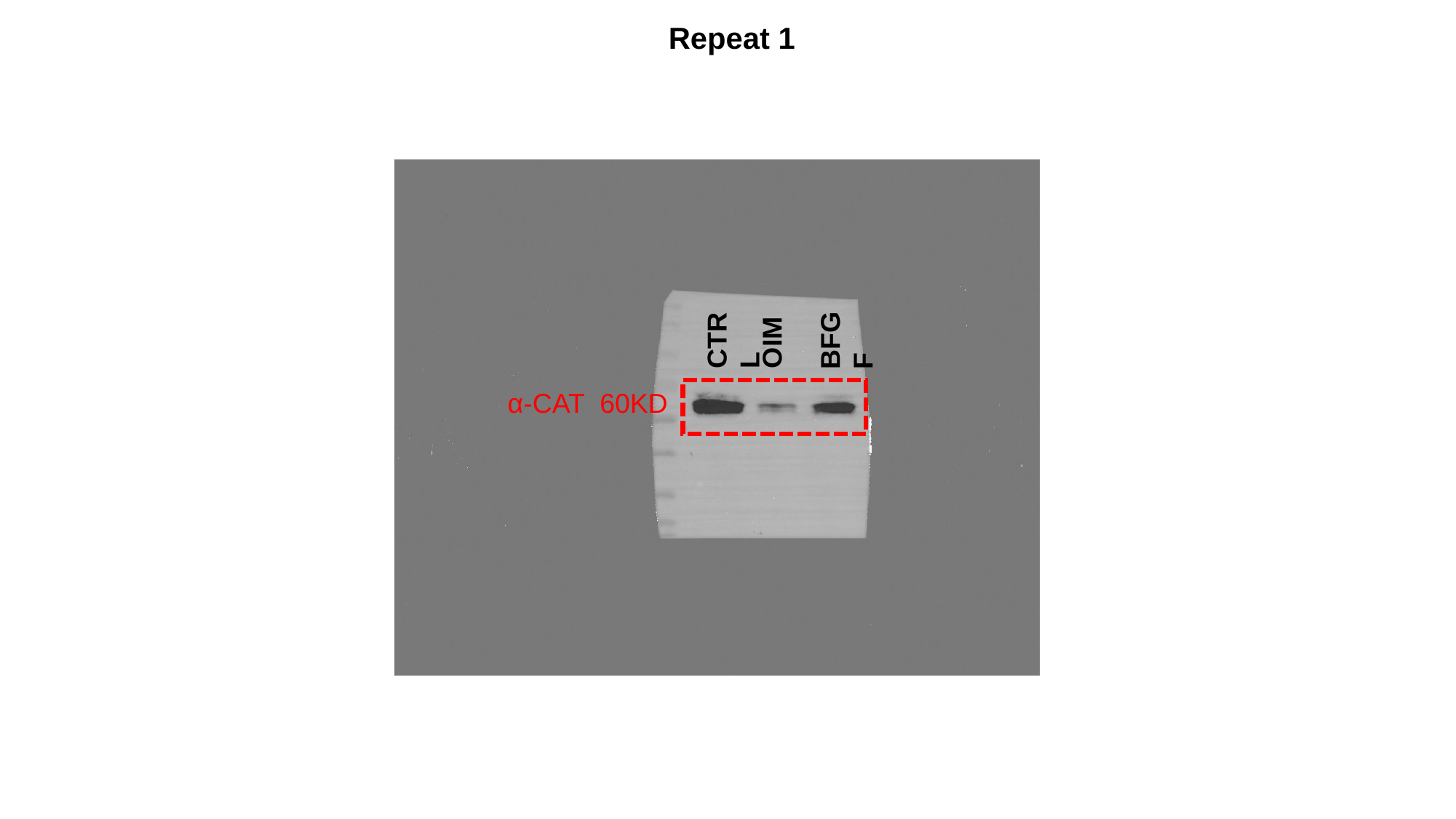

Repeat 1
OIM
CTRL
BFGF
α-CAT 60KD

## Slide 12
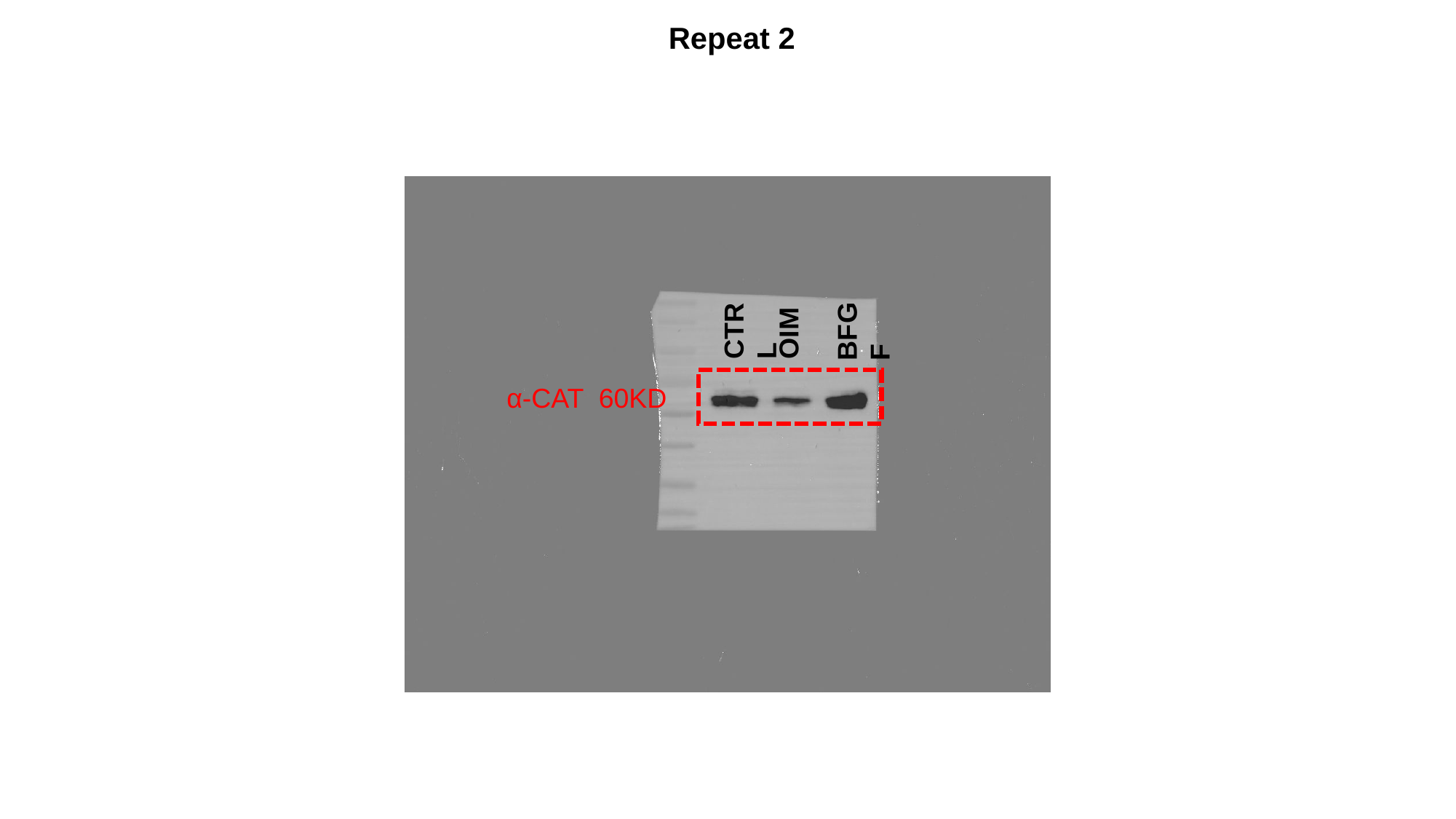

Repeat 2
OIM
CTRL
BFGF
α-CAT 60KD

## Slide 13
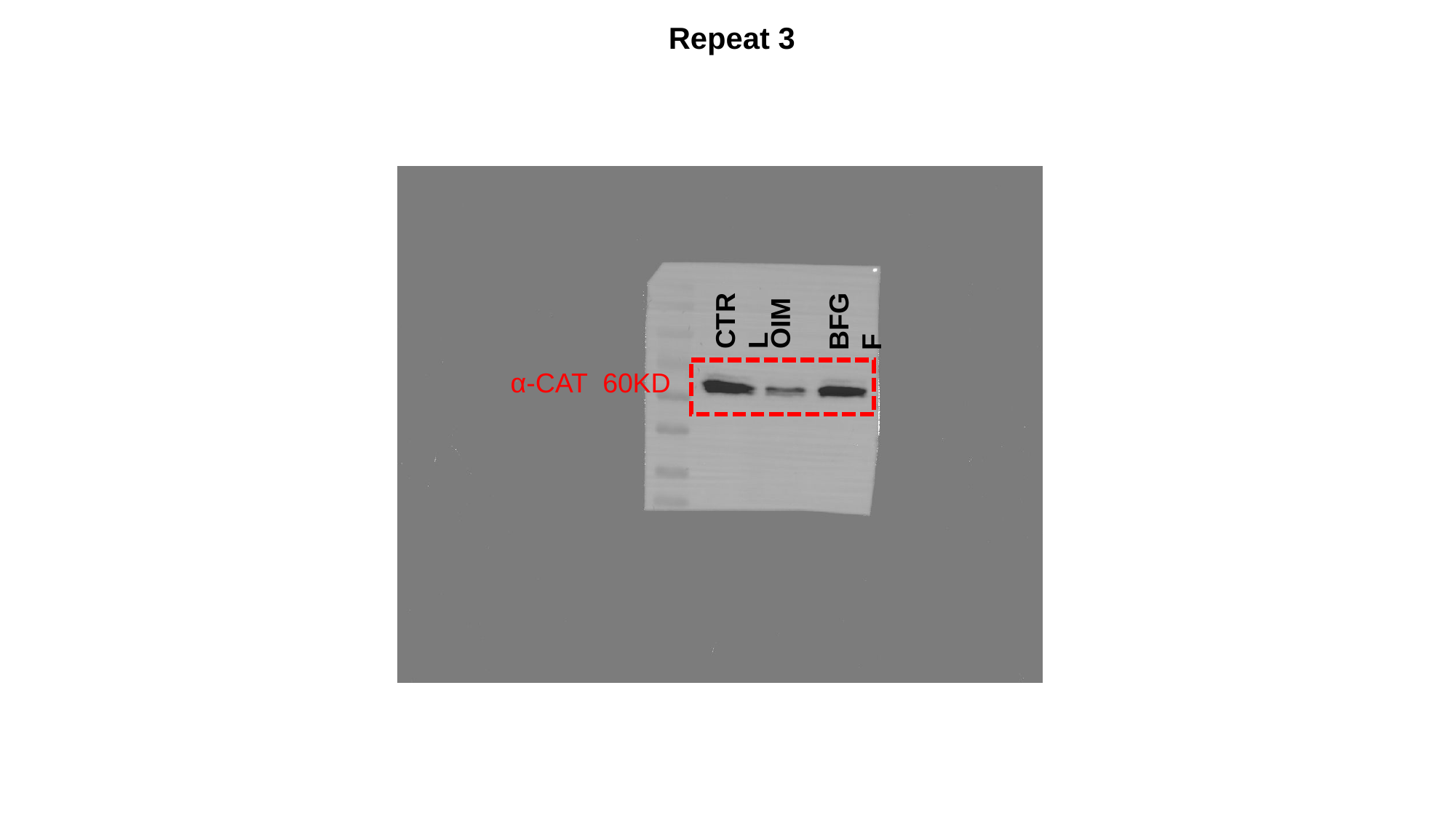

Repeat 3
OIM
CTRL
BFGF
α-CAT 60KD

## Slide 14
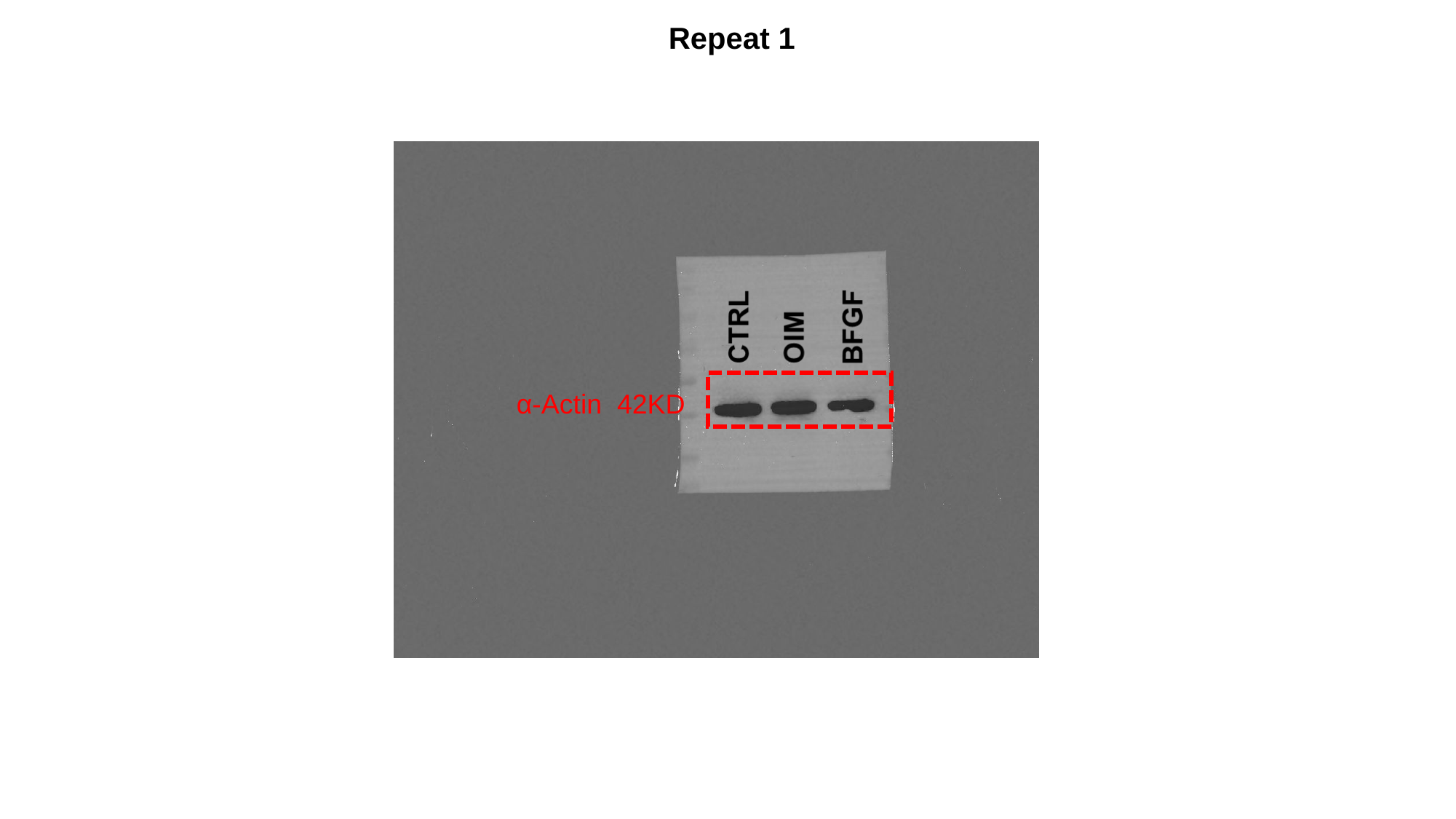

Repeat 1
α-Actin 42KD

## Slide 15
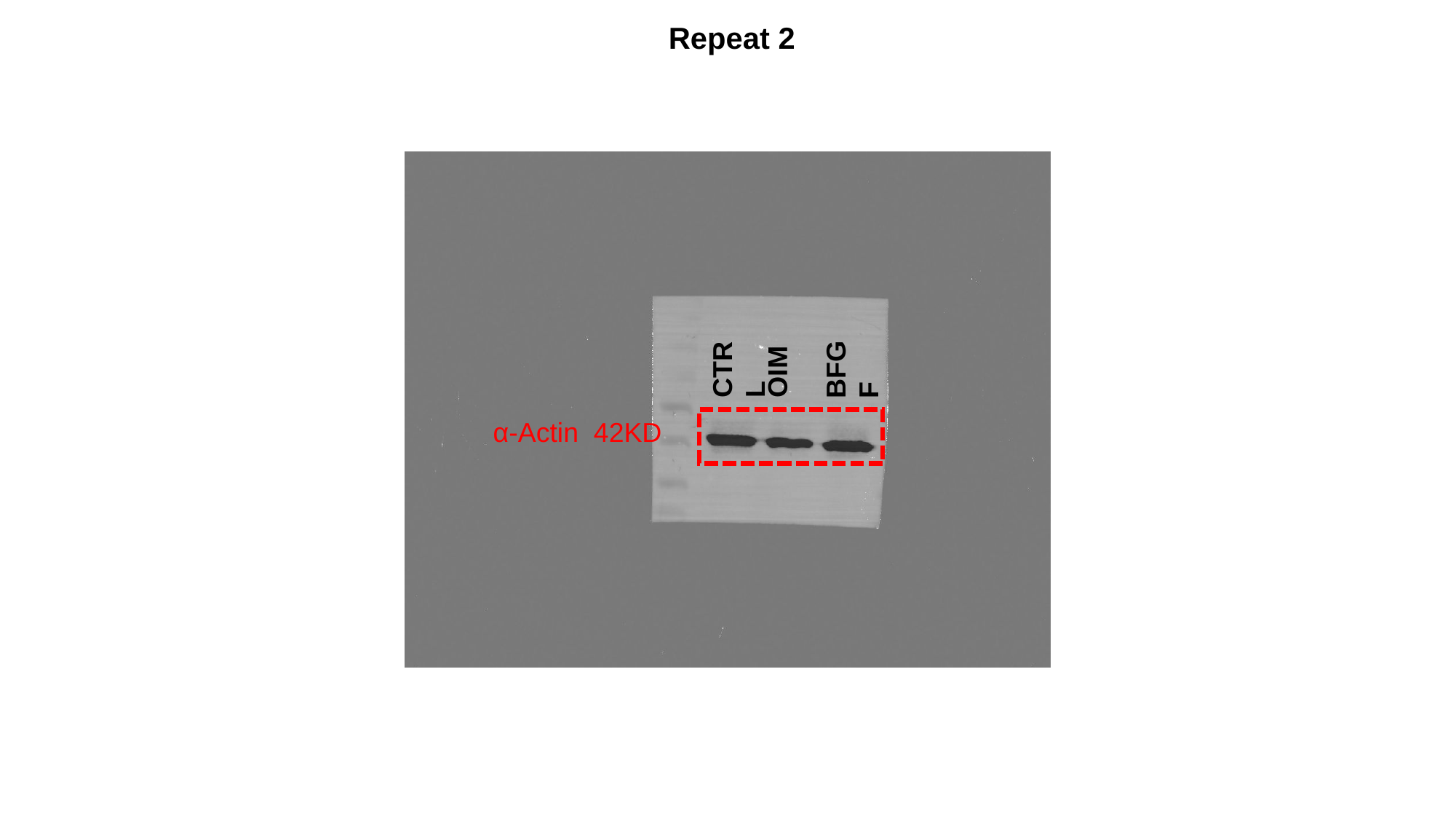

Repeat 2
OIM
CTRL
BFGF
α-Actin 42KD

## Slide 16
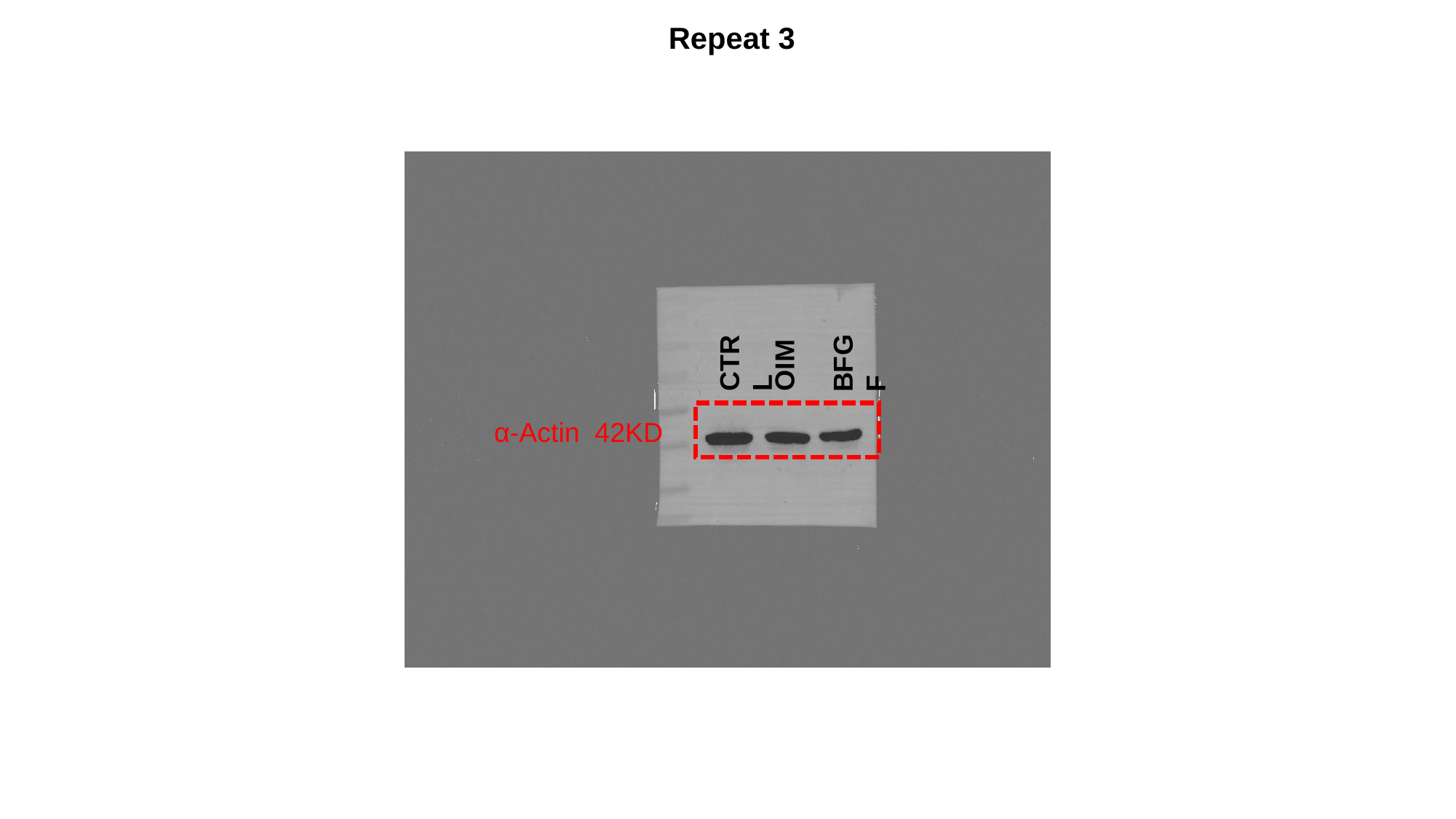

Repeat 3
OIM
CTRL
BFGF
α-Actin 42KD
